# TNF-α and Tumor Lysate Promote the Maturation of Dendritic Cells for Immunotherapy for Advanced Malignant Bone and Soft Tissue Tumors

**DOI:** 10.1371/journal.pone.0052926

**Published:** 2012-12-21

**Authors:** Shinji Miwa, Hideji Nishida, Yoshikazu Tanzawa, Munetomo Takata, Akihiko Takeuchi, Norio Yamamoto, Toshiharu Shirai, Katsuhiro Hayashi, Hiroaki Kimura, Kentaro Igarashi, Eishiro Mizukoshi, Yasunari Nakamoto, Shuichi Kaneko, Hiroyuki Tsuchiya

**Affiliations:** 1 Department of Orthopaedic Surgery, Kanazawa University School of Medicine, Kanazawa, Japan; 2 Department of Disease Control and Homeostasis, Kanazawa University School of Medicine, Kanazawa, Japan; UCLA, United States of America

## Abstract

**Background:**

Dendritic cells (DCs) play a pivotal role in the immune system. There are many reports concerning DC-based immunotherapy. The differentiation and maturation of DCs is a critical part of DC-based immunotherapy. We investigated the differentiation and maturation of DCs in response to various stimuli.

**Methods:**

Thirty-one patients with malignant bone and soft tissue tumors were enrolled in this study. All the patients had metastatic tumors and/or recurrent tumors. Peripheral blood mononuclear cells (PBMCs) were suspended in media containing interleukin-4 (IL-4) and granulocyte-macrophage colony stimulating factor (GM-CSF). These cells were then treated with or without 1) tumor lysate (TL), 2) TL + TNF-α, 3) OK-432. The generated DCs were mixed and injected in the inguinal or axillary region. Treatment courses were performed every week and repeated 6 times. A portion of the cells were analyzed by flow cytometry to determine the degree of differentiation and maturation of the DCs. Serum IFN-γ and serum IL-12 were measured in order to determine the immune response following the DC-based immunotherapy.

**Results:**

Approximately 50% of PBMCs differentiated into DCs. Maturation of the lysate-pulsed DCs was slightly increased. Maturation of the TL/TNF-α-pulsed DCs was increased, commensurate with OK-432-pulsed DCs. Serum IFN-γ and serum IL-12 showed significant elevation at one and three months after DC-based immunotherapy.

**Conclusions:**

Although TL-pulsed DCs exhibit tumor specific immunity, TL-pulsed cells showed low levels of maturation. Conversely, the TL/TNF-α-pulsed DCs showed remarkable maturation. The combination of IL-4/GM-CSF/TL/TNF-α resulted in the greatest differentiation and maturation for DC-based immunotherapy for patients with bone and soft tissue tumors.

## Introduction

Dendritic cells (DCs) play a pivotal role in the immune system, bridging the innate and adaptive immune responses [1]–[3]. DCs are critical professional antigen-presenting cells (APCs), crucially important in the capture, processing and presenting of tumor antigens to tumor-specific T cells [4], [5]. Necrotic tumor debris contains endogenous “danger signals”, required for the activation and maturation of APCs. DCs reach the damaged tissue, take up tumor antigens in the context of inflammation and abundant cytokines, and undergo a change in phenotype characterized by the upregulation of cell surface markers, such as costimulatory, adhesion and integrin molecules. The activated APCs migrate to tumor-draining lymph nodes where they present tumor antigens on major histocompatibility complex (MHC) molecules, as well as costimulatory signals, to resting tumor-specific T cells, inducing their activation [6]. Activated effector CD4^+^ T cells can attack MHC class II^+^ tumors and provide help to a variety of antitumor effector cells, including CD8^+^ cytotoxic T cells, B cells and macrophages [7]–[9].

There are a number of experimental and clinical studies about DC-based immunotherapies [10]–[12]. In the study of malignant bone and soft tissue tumors, there are some reports concerning DC-based immunity. Joyama reported that DCs inhibit primary tumors and metastatic tumors in an osteosarcoma model using LM8 [13]. Kawano reported that frozen tumor and DCs inhibit metastatic tumors, and increase IFN-γ and the migration of CD8-positive cells to the metastatic site in the osteosarcoma model [14]. These reports suggest that DC-based immunotherapy may be effective in the treatment of sarcoma. However, there are few reports about DC-based immunotherapy for malignant bone and soft tissue tumor.

DCs are present in peripheral blood. However, the concentration of DCs in the blood cells is too low to make their collection practical for clinical applications [15]. Instead, DCs are manufactured in either hematopoietic stem cells or peripheral blood monocytes [15], [16]. DCs are most frequently produced from peripheral blood mononuclear cells (PBMCs). Immature DCs are generated from PBMCs by suspension in media containing interleukin-4 (IL-4) and granulocyte-macrophage colony stimulating factor (GM-CSF) for several days. In the steady state, nonactivated (immature) DCs present self-antigens to T cells, which leads to tolerance [17], [18]. Conversely, activated (mature) DCs initiate antigen-specific immunity, resulting in T-cell proliferation and differentiation into helper and effector cells. DCs are also important in launching humoral immunity, partly because of their capacity to directly interact with B cells and to present unprocessed antigens. Therefore, the maturation of DCs is important for DC-based immunotherapy. Immature DCs (iDC) can be matured using various exogenous stimuli, including cytokines, growth factors, costimulatory molecules and inflammatory signals. Although various methods of generating DCs have been reported, the methods for differentiation and maturation of DCs are controversial. In this study, we developed methods for DC maturation using tumor necrotic factor alpha (TNF-α). The aim of our study was to generate and to investigate the differentiation and maturation of DCs. DCs were generated using various stimuli on PBMCs from patients with advanced malignant bone and soft tissue tumors.

## Patients and Methods

### Patients

Inclusion criteria were as follows: radiological and pathological diagnosis of malignant bone and soft tissue tumor, surgically resected tumor, age ≥6 years old, informed consent, and performance status (PS) ≤2.

Exclusion criteria included: severe cardiac, renal, pulmonary, hematological or other systemic disease associated with a discontinuation risk; chemotherapy or radiation therapy within four weeks; immunological disorders including splenectomy and radiation of the spleen; corticosteroid or anti-histamine therapy; or difficulty in follow-up.

This study protocol was approved by the Institutional Review Board of the Kanazawa University Graduate School of Medical Science, Kanazawa, Japan. This study complied with ethical standards outlined in the Declaration of Helsinki. Written informed consent was obtained from all patients and/or their parents before entry of the patients into this study. In cases that the patients were under twenty years old, the informed consent was obtained from all the parents.

### Preparation of Tumor Lysates

Tumor lysates (TL) were prepared from tumor tissues obtained from resected tumors. A portion of the tumor tissues (1 cm^3^) was frozen in liquid nitrogen for 20 min, and then thawed at room temperature for 20 min. The freeze-thaw cycle was repeated two times. Then, the tumor tissues were stirred and centrifuged, and supernatants were collected through a 0.2 μm filter. The amounts of protein in TL were evaluated by protein assay using a Bio-Rad protein assay kit (Bio-Rad, Tokyo, Japan) and stored at –20°C for later use.

### Preparation of Autologous Dendritic Cells (DCs)

**Figure 1:**
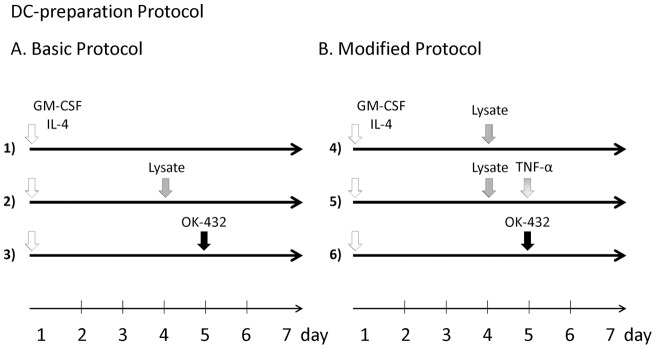
Preparation of DC from peripheral blood monocytes. A. Basic DC-preparation Protocol. DCs were generated from peripheral blood (50 ml) collected from study patients. Mononuclear cells were isolated by density gradient centrifugation on Lymphoprep. Interphase cells were removed, washed twice in phosphate-buffered saline (PBS), and suspended in CellGro. After adherence for 2 hours, adherent cells were cultured in media containing IL-4 (50 ng/ml) and GM-CSF (100 ng/ml). On days 4 and 5, the cultures were additionally supplemented with or without tumor lysate (TL) (100 μg/ml) and OK-432 (0.1 KE/ml). **B. Modified DC-preparation Protocol.** TNF-α was added to the protocol of tumor lysate and OK432. Similar to the first DC preparation protocol, the cells were cultured in a medium containing IL-4 and GM-CSF. On days 4 and 5, the cultures were additionally supplemented with or without tumor lysate (TL) (100 μg/ml), rhTNF-α (100 ng/ml), and OK-432 (0.1 KE/ml).

As previously reported, DCs were generated from blood monocyte precursors [19], [20]. Briefly, PBMCs from each patients were isolated by centrifugation in LymphoprepTM Tubes (Nycomed, Roskilde, Denmark). For generating DCs, PBMCs were plated in six-well tissue culture dishes (Costar, Cambridge, MA, USA) at 1– 4×10^7^ cells in 2 ml per well and allowed to adhere to plastic for 2 h. Adherent cells were cultured in serum-free media (GMP CellGro® DC Medium; CellGro, Manassas, VA, USA) with 50 ng/ml recombinant human IL-4 (GMP grade; CellGro®) and 100 ng/ml recombinant human granulocyte–macrophage colony-stimulating factor (GM-CSF) (GMP grade; Cell- Gro®) for 7 days. DC maturation was performed with various combinations of stimuli: TL, TL/TNF-α, or OK432 (Bicibanil, Chugai Pharmaceutical Co. Ltd, Tokyo, Japan) (Figure 1). Basic DC-preparation protocol comprised of TL and OK-432. Modified DC-preparation protocol comprised of TL, TNF-α and OK-432. On day 7, the generated DCs were harvested and mixed for injection, and 5×10^6^ cells were reconstituted in 1 ml normal saline. The DCs were injected in the inguinal or axillary region which the original tumor was closer. Treatment courses were performed every week and repeated 6 times. During the course of treatment, a portion of the cells were analyzed by flow cytometry to determine the degree of differentiation and maturation of the DCs.

### Flow Cytometry Analysis

DC preparation was assessed by staining with the following monoclonal antibodies: (MoAb) for 30 min on ice, antilineage cocktail 1 (lin-1; CD3, CD14, CD16, CD19, CD20 and CD56)-fluorescein isothiocyanate (FITC), anti-HLA-DR peridinin chlorophyll protein (PerCP) (L243), anti-CD14-allophycocyanin (APC) (MÖP9), anti-CD11c-APC (S-HCL-3), anti-CD123-phycoerythrin (PE) (9F5) (BD PharMingen, San Diego, CA, USA), anti-CD80-PE (MAB104), anti-CD83-PE (HB15a) and anti-CD86-PE (HA5.2B7) (Beckman Coulter, Fullerton, CA, USA). Cells were analyzed on a FACS Calibur^TM^ flow cytometer. Data analysis was performed with CELLQuestTM software (Becton Dickinson, San Jose, CA, USA). To determine the degree of differentiation and maturation of DCs, %CD14^–^HLA-DR^+^, %CD80, %CD83, and %CD86 were measured.

### Immune Response

Blood samples were collected from patients before surgery and one and three months after surgery. IFN-γ and IL-12 were measured in order to determine the magnitude of the immune response following the DC-based immunotherapy.

### Overall survival and progression free survival following the DC-based immunotherapy

Overall survival and progression free survival were evaluated by Kaplan-Meier method. Survival was defined as the time from the date of end of DC-based immunotherapy to the date of last follow-up or death due to any cause. Progression free was defined as the time from the date of end of DC-based immunotherapy to the date of last follow-up or tumor progression.

### Statistical Analysis

Results are expressed as mean ± SEM. Data were analyzed using one-way analysis of variance (ANOVA) test. Any P-values less than 0.05 were considered statistically significant. Statistical analyses were performed on a personal computer with the statistical package ystat2000 software.

## Results

### Patients

Thirty-one patients were included in this study. All the patients had metastatic and/or recurrent tumors. There were 14 males and 17 females, with a mean age of 36.3 years (8−64, Table 1). There were 16 patients with bone tumors (13 osteosarcoma and 3 chondrosarcoma,) and 15 patients with soft tissue tumors (3 clear cell sarcoma, 3 synovial sarcoma, 3 malignant fibrous histiocytoma (MFH), 2 leiomyosarcoma, 1 Ewing's sarcoma, 1 liposarcoma, 1 alveolar soft part sarcoma (ASPS), and 1 angiosarcoma). None has experienced an adverse event during and after the treatment course.

**Table 1:** Characteristics of study patients.

No.	Gender	Age	Diagnosis	Metastatic tumor	Recurrence	primary	Protocol
1	M	26	Osteosarcoma	Lumbar, lung	+	Tibia	Basic
2	M	15	Osteosarcoma	Lung	+	Tibia	Basic
3	F	9	Osteosarcoma	Lung, lumbar	–	Femur	Basic
4	M	31	Chondrosarcoma	Lung	–	Tibia	Basic
5	F	53	Leiomyosarcoma	Lung	+	Inguen	Basic
6	F	19	Osteosarcoma	Lung	–	Femur	Basic
7	F	17	Osteosarcoma	Pelvis, humerus, lumbar, lung	–	Femur	Basic
8	F	49	Osteosarcoma	Lung, tibia, brain	–	Femur	Basic
9	F	61	Clear cell sarcoma	–	+	Foot	Basic
10	F	40	ASPS	Lung	–	Hand	Basic
11	M	65	Leiomyosarcoma	–	+	Thigh	Basic
12	M	24	Osteosarcoma	Lung	–	Femur	Basic
13	M	45	Osteosarcoma	Lung, femur	+	Pelvis	Basic
14	M	19	Osteosarcoma	Lung, brain	–	Femur	Basic
15	M	59	MFH	Lung	+	Tibia	Basic
16	M	29	Angiosarcoma	Lung	–	Lower leg	Basic
17	M	43	Chondrosarcoma	Lung, calcaneus	–	Tibia	Basic
18	F	43	Clear cell sarcoma	Lung	–	Lower leg	Basic
19	F	26	Clear cell sarcoma	Thigh, inguen	–	Lower leg	Basic
20	M	59	Synovial sarcoma	lung	–	Lower leg	Basic
21	M	30	Osteosarcoma	Lung	–	Femur	Basic
22	F	39	Chondrosarcoma	–	+	Pelvis	Modified
23	M	37	Liposarcoma	Lung	–	Thigh	Modified
24	F	27	Osteosarcoma	Lung	–	Pelvis	Modified
25	F	41	Synovial sarcoma	Lung	–	Back	Modified
26	F	24	Synovial sarcoma	Lung	–	Inguen	Modified
27	M	64	MFH	–	+	Back	Modified
28	F	8	Ewing's sarcoma	Lung	–	Calf	Modified
29	M	31	Osteosarcoma	Lung	–	Femur	Modified
30	F	64	MFH	Lung	–	Shoulder	Modified
31	F	28	Osteosarcoma	Lung	–	Pelvis	Modified

Protocol: DC-preparation protocol; MFH, malignant fibrous histiocytom; ASPS, alveolar soft part sarcoma; MPNST, malignant peripheral nerve sheath tumor.

### Differentiation of DCs

To determine the degree of differentiation, %CD14^–^HLA-DR^+^ was measured by flow cytometry. In total, 52.5±1.2% of cells were classified as CD14^–^HLA-DR^+^ cells (Table 2). The cells treated with IL-4, GM-CSF, and TL showed more differentiation than the cells treated with IL-4 and GM-CSF only (not significant). The cells treated with IL-4, GM-CSF, and OK-432 showed more differentiation than the cells treated with the other protocols (P<0.01). Furthermore, the cells treated with IL-4, GM-CSF, TL, and TNF-α showed more differentiation than the cells treated with IL-4, GM-CSF, and lysate alone (P<0.05). These results suggest that TL, TNF-α and OK-432 promoted the differentiation of DCs.

**Table 2:** Comparison of %CD14^–^HLA-DR^+^ cells by DC maturation cocktails.

Group	DC-preparation protocol		%CD14^–^HLA-DR^+^
Control	1)	IL-4, GM-CSF	45.4±2.5%
TL	2),4)	IL-4, GM-CSF + TL	50.0±1.8%
TNF-α	5)	IL-4, GM-CSF + TL + TNF-α	55.6±2.9%*
OK-432	3),6)	IL-4, GM-CSF + OK-432	60.1±1.9%**
		Total	52.5±1.2%

CD14: mononuclear cell marker, HLA-DR: dendritic cell marker, IL-4: interleukin-4, GM-CSF: granulocyto-macrophage colony stimulating factor, TL: tumor lysate.

Data±SEM *P<0.05, **P<0.01 versus control group.

### Maturation of DCs

**Figure 2:**
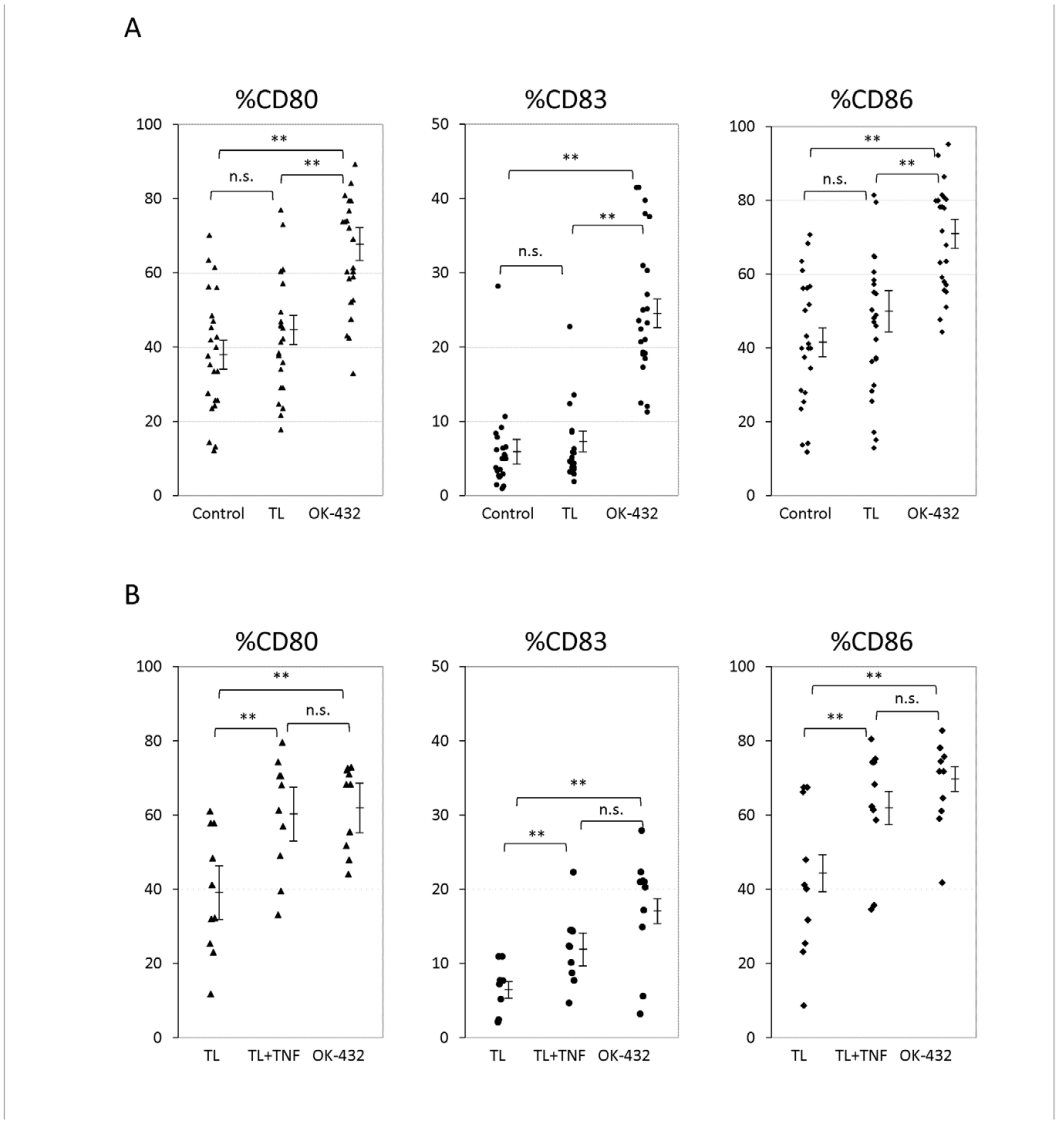
Comparison of %CD80, %CD83, and %CD86 by DC maturation cocktails. TL: tumor lysate, TNF: tumor necrosis factor-α, ** P<0.01, n.s.: not significant **A. Basic DC-preparation Protocol.** Cells treated with tumor lysate (TL) showed slightly higher maturation than control cells (not significant). Cells treated with OK-432 showed greater maturation (P<0.01). **B. Modified DC-preparation Protocol.** Cells treated with a combination of tumor lysate (TL) and TNF-α (TNF) showed markedly higher maturation than cells treated with TL (P<0.01).

To determine the effects of DC maturation by TL, OK-432, and the combination of TL and TNF-α, %CD80, %CD83, and %CD86 were measured (Figure 2).

The effects of DC maturation by TL and OK-432 (basic DC preparation protocol) were analyzed (Figure 2–A). The %CD80 of the control group, TL group, and OK-432 group were 38.3±3.4, 42.4±3.2, and 64.2±3.1%, respectively. The %CD83 of the control group, TL group, and OK-432 group were 5.9±1.2, 6.2±1.0, and 25.1±1.9%, respectively. The %CD86 of the control group, TL group, and OK-432 group were 41.7±3.6, 45.5±3.9, and 69.9±3.0%, respectively. OK-432 group showed significant increase of %CD80, %CD83, %CD86 comparing with the other groups (P<0.01). However, there was no significant difference between TL group and control group. These results indicate that TL slightly induced the maturation of the DCs and that OK-432 markedly induced the maturation of DCs.

The effects of DC maturation by the combination of TL and TNF-α, or OK-432 (modified DC preparation protocol) were analyzed (Figure 2–B). The %CD80 of the TL group, TL + TNF-α (TNF) group, and OK-432 group were 39.1±5.3, 60.3±4.9, and 62.5±3.6%, respectively. The %CD83 of TL group, TL + TNF group, and OK-432 group were 4.5±1.1, 12.2±1.5, and 17.5±2.4%, respectively. The %CD86 of TL group, TL + TNF group, and OK-432 group were 42.0±6.5, 62.5±5.1, and 68.2±3.8%, respectively. Both of TL + TNF group and OK-432 group showed significant increase of %CD80, %CD83, %CD86 comparing with TL groups (P<0.01). These results indicate that the combination of TL and TNF-α markedly induced the maturation of DCs.

### Immune Responses

**Figure 3:**
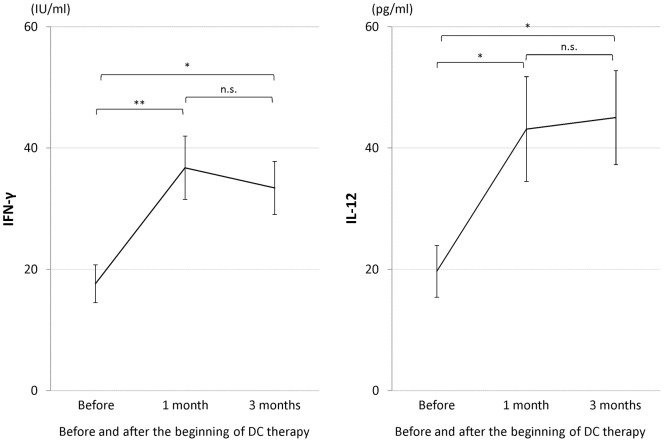
Serum IFN-γ and serum IL-12 before and after DC therapy. * P<0.05, ** P<0.01, n.s.: not significant.

Serum IFN-γ and serum IL-12 were compared prior to DC-based immunotherapy, and one and three months after DC injection (Figure 3). Serum IFN-γ before DC injection, and one and three months after the injection was 17.6±3.1, 36.8±5.2, and 33.4±4.4 IU/ml, respectively. Serum IL-12 before DC injection, and one and three months after the injection was 19.7±4.3, 43.1±8.6, and 45.0±7.8 pg/ml, respectively. Both of IFN-γ and IL-12 showed significant increases after DC injection. These results indicate that our DC therapy induced the activation of immune responses one month after the therapy, and that the activated immune response is maintained for at least three months.

### Overall survival and Progression free survival

**Figure 4:**
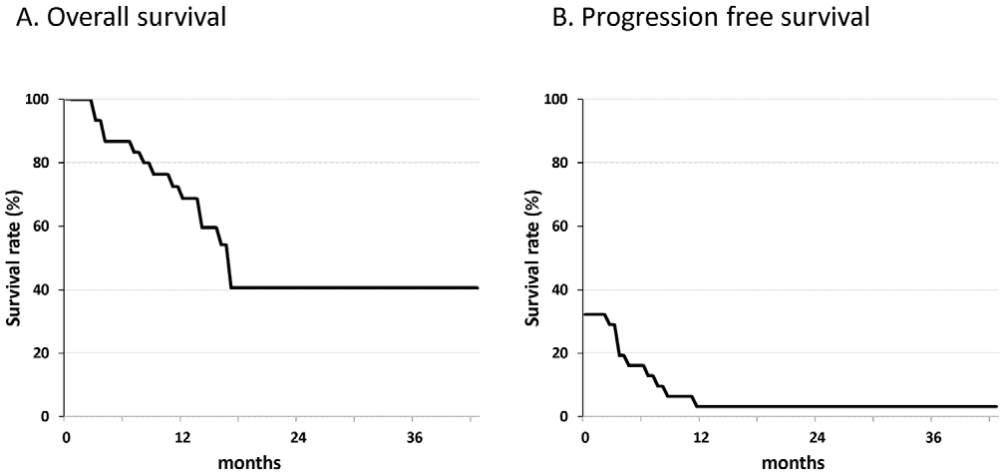
Kaplan-Meier analysis of overall survival and progression free survival distributions for 31 patients with advanced malignant bone and soft tissue tumors.

The mean follow-up of all patients was 14.9 months (range 2– 43 months). Of the 31 patients, 17 patients were alive and 1 patient was progression free at the time of the final follow-up. Overall survival rates and progression free survival rates of the patients at 3 years were 68.7% and 3.2%, respectively (Figure 4).

## Discussion

The introduction of chemotherapy dramatically improved the treatment outcome for patients with primary bone and soft tissue sarcoma [21]. The standard surgery for bone and soft tissue sarcoma before the introduction of chemotherapy was limb amputation [21]. Consequently, the prognosis of patients with bone and soft tissue sarcoma was very poor before the introduction of chemotherapy. The most important prognostic factor before the introduction of chemotherapy was the metastatic lesions that existed before limb amputation. Unlike surgery and radiotherapy, chemotherapy shows systemic antitumor effects, which affect primary tumors and metastatic tumors. However, the continuation of chemotherapy causes many complications, such as renal failure, heart failure, and neuropathy. In cases with local recurrence or metastatic tumors, complications make it difficult to continue the chemotherapy course. Therefore, further systemic treatment of sarcoma is required an improved prognosis of patients with bone and soft tissue tumors. Recently, many experimental and clinical studies concerning immunotherapy for the treatment of malignant tumors have been reported [12]. Additionally, some patients showed a possible tumor suppressive effect of immunotherapy, indicating that this could be a promising approach in the treatment of patients with refractory malignant tumors.

Immunotherapy is classified as either non-specific immunotherapy or tumor specific immunotherapy [22], [23]. Non-specific immunotherapy includes cytokine therapy, lymphokine-activated killer cells therapy, natural killer cell therapy, ERM activated killer cell therapy, and CD3-activated T cell therapy. Tumor-specific immunotherapy includes DC-based immunotherapy, cytotoxic T lymphocyte therapy, and peptide-vaccine therapy.

DCs play a critical role in T cell priming, as well as direct and cross-priming [12]. In the steady state, nonactivated (immature) DCs present self-antigens to T cells, which leads to tolerance through different mechanisms. Once activated (mature), antigen-loaded DCs promote T cell proliferation and differentiation into helper and effector cells. DCs are also important in launching humoral immunity. This is partly due to their capacity to directly interact with B cells and to present unprocessed antigens. DCs demonstrate tumor-specific antitumor effects by presenting tumor-specific antigens to immune cells. Furthermore, DCs play a pivotal role in the immunity to tumors. Once a dendritic cell takes up tumor antigens, the dendritic cell will present the tumor antigens to 100– 5,000 lymphocytes. Therefore, DC-based immunotherapy seems to be an efficient treatment for malignant tumors. However, the population of DCs in PBMCs is 0.2% [24], which is too low to make their collection practical for clinical applications [15]. Methods of collection or generation of DCs are required in order to use DC-based immunotherapy clinically. To date, PBMCs-derived DCs have been commonly used for DC-based immunotherapy.

IL-4 and GM-CSF are commonly used for the differentiation of DCs. Nakamoto et al. [25] reported that 45% of PBMCs in patients with hepatocellular carcinoma were differentiated into DCs by the treatment with IL-4 and GM-CSF. This study showed that 52.5% of PBMCs in patients with bone and soft tissue tumors were differentiated into DCs by IL-4 and GM-CSF. Furthermore, DC maturation requires stimuli such as IL-1β, TNF-α, IL-6, or prostaglandin E_2_. Although various DC maturation cocktails have been reported, there is no gold standard method for DC maturation for DC-based immunotherapy. OK-432 (Picibanil), a penicillin-killed Streptococcus pyogenes, is reported to have potent immunomodulation properties in cancer treatment [26]. Hovden reported that OK-432 induces the maturation and migration of DCs and stimulates the secretion of Th-1 type cytokines [27]. Our results show that OK-432 had a strong effect on DCs-differentiation and DC maturation. Although OK-432 has strong effects on DC-differentiation and DC maturation, OK-432-pulsed DCs are not able to activate tumor antigen-specific immunity. Conversely, tumor antigen-pulsed DCs can induce a tumor-specific immune response. Hsu *et al.* [10] reported that DC pulsed with a tumor antigen could elicit specific tumor-reactive T cells, and have clinical efficacy in patients with lymphoma. As with other malignancies, TL-pulsed DCs seem to be effective in the treatment of sarcoma. Our results showed that the TL slightly promoted differentiation and maturation of DCs. The effect of promoting DC-differentiation and DC maturation suggests the existence of tumor-specific antigens in many malignant bone and soft tissue tumors. Additionally, our results showed that TNF-α has a strong effect on promoting DC maturation. The TL/TNF-α pulsed DCs showed 1.6– 1.7 times greater maturation than the TL-pulsed DCs. Although TL-pulsed DCs can present tumor antigens, the TL-pulsed DCs showed only slight maturation. TNF-α promotes the maturation of TL-pulsed DCs. The well-differentiated and well-matured DCs generated by our DCs-generation protocols are considered to have strong tumor antigen-specific effects.

DC-based immunotherapy has been generally ineffective in promoting tumor rejection. The failure may be attributed to the use of insufficiency matured or activated DCs. In patients with advanced and metastatic cancer, immunity is generally suppressed by factors produced by tumors and tumor infiltrating cells such as regulatory T cells [28]. In order to overcome the immunosuppressive circumstances around tumors, inductions of sufficiently activated DCs, which highly express MHC molecules, CD80, CD86, and IL-12, are required. The existence of matured DCs around tumors has a great importance in antitumor immune response and prognostic significance [29], [30]. Ménard et al. reported that levels of IFN-γ significantly correlate with progression-free survival [31].

Therefore, evaluations of the DCs-maturation and DCs-related cytokines are important in the DC-based immunotherapy. In this study, levels of IFN-γ and IL-12 were measured before and after the immunotherapy. The immunological responses are commonly evaluated by IL-12 and IFN-γ because these cytokines reflect the activation of the DCs [14], [32]–[35]. Furthermore, it is reported that the existence of tumors does not influence the levels of the serum IL-12 and IFN-γ [32]. IL-12 secreted by DCs acts on IFN-γ production by Th1 cells. IFN-γ activates natural killer (NK) cells and cytotoxic T lymphocytes (CTL) that contribute to optimal antitumor immunity. Although chemotherapy shows rapid effects on tumors, DC-based immunotherapy shows slow tumor regression. The immune response is highest at 8– 10 weeks after DC-injection [36]. This study showed significant elevations of IFN-γ and IL-12 at one and three months after DC-based immunotherapy. However, a limitation of this study is the lack of data about serum IFN-γ and IL-12 in placebo group. Therefore, it seems that tumor progression influenced the increase of serum IFN-γ and IL-12. There are some reports about the correlation between tumor progression and serum cytokine levels [37], [38]. Tsuboi et al. [37] reported that mean serum IL-12 level in patients with esophageal carcinoma (n = 70) was significantly higher than that in healthy volunteers (15). The levels of serum IL-12 correlated with tumor growth and progression, although the there was no significant correlation between serum IL-12 level and tumor progression. Morreti et al. [38] reported the difference of the serum IL-12 and IFN-γ levels in control population (n = 45), patients with localized melanoma (n = 11), and patients with metastatic melanomas (n = 34). Mean serum IL-12 level of the patients with localized melanomas was lower than that of the patients with metastatic melanomas. Mean serum IFN-γ level of patients with localized melanoma was higher than that of patients with metastatic melanomas, although there is no significant difference. In our study, serum IL-12 and IFN-γ showed significant increases which are much higher than the influence of tumor progression. These results suggest that the increase of the serum IL-12 and IFN-γ levels were influenced by the DC-based immunotherapy.

## Conclusions

DCs-based immunotherapy is a promising approach for the treatment of malignant tumors. In this study, about 50% of PBMCs were differentiated into DCs, and markedly matured by the combination of GM-CSF, IL-4, TL and TNF-α. Our results contribute to the development of DC-based immunotherapy for malignant bone and soft tissue tumors.
