# Correction: TNF-α and Tumor Lysate Promote the Maturation of Dendritic Cells for Immunotherapy for Advanced Malignant Bone and Soft Tissue Tumors

**DOI:** 10.1371/annotation/7c0f14e6-cfba-4f5f-b93d-1a149eaeec96

**Published:** 2013-10-24

**Authors:** Shinji Miwa, Hideji Nishida, Yoshikazu Tanzawa, Munetomo Takata, Akihiko Takeuchi, Norio Yamamoto, Toshiharu Shirai, Katsuhiro Hayashi, Hiroaki Kimura, Kentaro Igarashi, Eishiro Mizukoshi, Yasunari Nakamoto, Shuichi Kaneko, Hiroyuki Tsuchiya

In the section titled, "Overall survival and Progression free survival" within the Results Section the value of overall survival of patients at 3 years was incorrectly described as 68.7%. The correct value is 40.6%. 

